# Mechanisms of Change in Cognitive‐Behavioral Therapy for Adults With Binge‐Eating Disorder: A Dynamic Structural Equation Modeling Approach

**DOI:** 10.1002/eat.24469

**Published:** 2025-05-21

**Authors:** Ricarda Schmidt, Danielle Schewe, Stephan Herpertz, Stephan Zipfel, Brunna Tuschen‐Caffier, Hans‐Christoph Friederich, Andreas Mayr, Martina de Zwaan, Anja Hilbert

**Affiliations:** ^1^ Integrated Research and Treatment Center AdiposityDiseases, Behavioral Medicine Research Unit, Department of Psychosomatic Medicine and Psychotherapy University of Leipzig Medical Center Leipzig Germany; ^2^ Department of Psychosomatic Medicine and Psychotherapy LWL‐University Bochum Germany; ^3^ Psychosomatic Medicine and Psychotherapy Medical University Hospital Tuebingen Tuebingen Germany; ^4^ Department of Clinical Psychology and Psychotherapy University of Freiburg Freiburg Germany; ^5^ Department of General Internal Medicine and Psychosomatics Medical University Hospital Heidelberg Heidelberg Germany; ^6^ Institute for Medical Biometry and Statistics Philipps‐University Marburg Marburg Germany; ^7^ Department of Psychosomatic Medicine and Psychotherapy Hannover Medical School Hannover Germany

**Keywords:** binge‐eating disorder, cognitive‐behavioral therapy, dietary restraint, mechanisms of change, overvaluation of shape and weight

## Abstract

**Objective:**

Cognitive‐behavioral therapy (CBT) is the most well‐established treatment for binge‐eating disorder (BED), but the mechanisms of change remain poorly understood. This study investigated in CBT for BED the effects of overvaluation of shape and weight and dietary restraint on subsequent objective binge‐eating episodes (OBEs).

**Method:**

In a multicenter randomized‐controlled trial, 84 patients diagnosed with full‐ or subsyndromal BED were offered 20 individual sessions of CBT over 4 months. Dynamic structural equation modeling (DSEM) was used to disentangle within‐ and between‐patient associations of overvaluation of shape and weight, dietary restraint, and OBEs.

**Results:**

Between the first and last week of therapy, there were significant reductions in overvaluation of shape and weight, dietary restraint, and OBEs. DSEM showed significant within‐patient effects of overvaluation of shape and weight on the subsequent number of OBEs. Weeks with lower overvaluation of shape and weight levels were followed by weeks with fewer OBEs. Although no within‐patient effect of dietary restraint on OBEs was found, within‐patient dietary restraint levels positively predicted subsequent overvaluation of shape and weight levels.

**Discussion:**

The findings suggest that reductions in overvaluation of shape and weight may precede improvements in binge eating during CBT for BED, supporting its role as a potential mechanism of change. While dietary restraint did not show a direct temporal link to binge eating, its association with overvaluation points to a potential indirect role. These results underscore the value of targeting cognitive features of BED in CBT and highlight the need for more temporally sensitive assessments in mechanisms research.

**Summary**

- This study explored factors influencing changes in cognitive‐behavioral therapy for binge‐eating disorder.
- The findings showed that reductions in overvaluation of shape and weight were linked to fewer binge‐eating episodes, highlighting its role as a mechanism of change.
- Changes in dietary restraint may also impact binge eating indirectly through overvaluation.
- These insights can help refine therapeutic approaches for patients with binge‐eating disorder.

## 1 Introduction

Binge‐eating disorder (BED) is the most prevalent eating disorder and is characterized by recurrent episodes of objective binge eating (OBEs), defined as the consumption of excessively large amounts of food accompanied by a sense of loss of control and occurring in the absence of regular compensatory behaviors aimed at preventing weight gain (American Psychiatric Association 2013). BED affects approximately 2.8% of women and 1.0% of men over the course of their lives (Galmiche et al. 2019) and is associated with an increased risk of obesity as well as a range of physical and mental comorbidities (Giel et al. 2022). While cognitive‐behavioral therapy (CBT) is the most well‐established treatment for BED, leading to abstinence from binge eating in approximately 50% of patients (Hilbert et al. 2019), the psychological processes through which this change occurs remain poorly understood (Grilo and Juarascio 2023). Traditionally, psychotherapy research has focused on establishing whether treatments are effective, yet much less attention has been devoted to understanding *how* and *why* they work. Outcome studies are essential, but they often leave a gap in knowledge regarding the underlying mechanisms that drive therapeutic improvement. Mechanisms of change are defined as theoretically grounded processes that are causally involved in symptom reduction and that are actively targeted and modified through treatment (Kazdin 2007). In contrast, mediators may statistically account for a treatment effect but are typically observed rather than directly manipulated. While broader psychological constructs, such as learning or motivation, may offer general explanations of therapeutic change, a more precise understanding of change processes requires greater conceptual specificity (Kazdin 2007).

While rapid response, commonly defined as a substantial reduction in OBEs by the fourth week of treatment, serves as a transdiagnostic indicator of early treatment progress (Hilbert et al. 2015), the cognitive‐behavioral maintenance model for eating disorders provides a disorder‐specific framework, highlighting overvaluation of shape and weight and dietary restraint as core maintaining factors addressed in CBT (Fairburn et al. 2003). Meta‐analytic evidence supports the superior efficacy of CBT over control conditions (e.g., waitlist, placebo, or non‐CBT interventions) in reducing overvaluation of shape and weight and dietary restraint from pre‐ to post‐treatment in patients with BED (Linardon 2018). Nevertheless, research into the mechanisms driving change in CBT for BED remains scarce, with most studies focusing on potential candidates such as reductions in dietary restraint (Bartholomay et al. 2024), dietary restriction (Sivyer et al. 2020), and impulsivity (Boswell et al. 2023). Findings regarding their roles as mechanisms underlying reductions in OBEs have been inconsistent, which may be partly attributable to methodological limitations, such as heterogeneous samples (e.g., combining BED and bulimia nervosa [BN]) or wide assessment intervals (e.g., every 4 weeks) that fail to capture short‐term symptom dynamics. Importantly, although overvaluation of shape and weight is recognized as a core maintaining factor in BED (Wang et al. 2019; Grilo et al. 2012), its potential role as a dynamic, time‐sensitive predictor of subsequent symptom change during CBT—and thus as a candidate mechanism of change—has not yet been empirically examined. While prior research has demonstrated that session‐by‐session improvements in overvaluation of shape and weight predicted next‐week changes in dietary restraint, binge eating, and compulsive exercise in 44 adults with BN undergoing CBT (Srivastava et al. 2023a), no comparable studies have been conducted in individuals with BED.

Findings on the relationship between dietary restraint—the cognitive effort to limit food intake to alter body weight or shape—and OBEs in interventional research have been mixed and, at times, controversial. In adults receiving cognitive‐behavioral self‐help (CBTgsh), lower baseline levels of dietary restraint were linked to greater reductions in OBEs (Anderson et al. 2020), but this association did not emerge in adolescents undergoing CBT for BED (Schmidt and Hilbert 2022). Early reductions in dietary restraint, specifically, by the fourth week of treatment, have been shown to predict subsequent improvements in binge eating in 154 patients with BN in a randomized controlled trial (RCT) comparing CBT and interpersonal psychotherapy (IPT) (Wilson et al. 2002). In contrast, among patients with BED receiving CBTgsh, minimal changes in dietary restraint throughout treatment did not predict post‐treatment abstinence from binge eating (Blomquist and Grilo 2011). Likewise, an ecological momentary assessment (EMA) study involving 112 individuals with BED found no association between changes in dietary restriction, operationalized as the number of hours spent fasting, and changes in binge‐eating frequency during integrative cognitive‐affective therapy or CBTgsh (Bartholomay et al. 2024). Taken together, and in light of the available experimental evidence, dietary restraint appears to function more as a moderating factor rather than as a direct causal driver of binge eating (Jansen 2025).

In sum, understanding the mechanisms of change for CBT in patients with BED remains complex and under‐researched. This study takes an important first step by examining the week‐to‐week relationships between overvaluation of shape and weight, dietary restraint, and OBEs in patients with BED during CBT. Repeated assessment of both potential mechanisms of change and outcome variables throughout treatment is crucial for advancing the understanding of the processes driving therapeutic change and for informing efforts to optimize treatment efficacy (Kazdin 2007). In line with previous findings (Linardon 2018), it was hypothesized that overvaluation of shape and weight, dietary restraint, and OBEs would decrease from pre‐ to post‐treatment. Changes in overvaluation of shape and weight were expected to predict subsequent changes in OBEs, with lower levels of overvaluation of shape and weight predicting fewer OBEs. In light of the conflicting findings regarding the role of dietary restraint, the effect of dietary restraint on subsequent OBEs was analyzed in an exploratory manner. Given the limited research on the relationship between overvaluation of shape and weight and dietary restraint within treatment, this relationship was also analyzed exploratorily. Considering that gender may be a moderator of treatment outcome in BED (Hilbert et al. 2019), a sensitivity analysis was conducted including women only. A second sensitivity analysis focused on modeling each potential mechanism of change separately to increase the understanding of the unique contribution of overvaluation of shape and weight and dietary restraint for OBEs.

## 2 Method

The present study is a secondary analysis of the multicenter INTERBED RCT (De Zwaan et al. 2017), comparing face‐to‐face CBT with Internet‐based guided self‐help for adults with BED. The recruitment of the study took place from August 2010 to December 2011, with final follow‐up assessments taking place in April 2014. Site‐specific institutional review boards provided ethical approval. Session‐wise assessments for overvaluation of shape and weight, dietary restraint, and OBEs were only available in the CBT arm; thus, data from the guided self‐help arm were excluded. Participants had to be ≥ 18 years old, German‐speaking, have a body mass index (BMI) between 27.0 and 40.0 kg/m^2^, and meet the DSM‐IV‐TR (American Psychiatric Association 2000) criteria for BED or subsyndromal BED, confirmed by the Eating Disorder Examination (EDE) (Fairburn and Cooper 1993; Hilbert and Tuschen‐Caffier 2016). Written informed consent and Internet access were required. Exclusion criteria were suicidal ideation, psychotic or bipolar disorder, current BN, substance abuse, medical conditions affecting weight or eating, ongoing psychotherapy, pregnancy, lactation, and use of antipsychotic or weight‐affecting medications. For this study, two out of 86 eligible patients were excluded due to missing week‐to‐week data. The final sample included 84 patients (72 women, 85.71%) aged 18–63 years (*M* = 42.55; SD = 12.11), with a mean BMI of 34.33 kg/m^2^ (SD = 3.95), and 38 patients (45.24%) with < 12 years of education.

### 2.1 Treatment

CBT was based on a validated manual (Hilbert and Tuschen‐Caffier 2010). Patients were offered twenty 50‐min individual sessions over 4 months, starting with motivational enhancement, followed by modules on eating behavior, body image, and stress, and ending with relapse prevention. Sessions were offered twice a week in the first month, weekly in months two and three, and biweekly in the fourth month. Double sessions were possible during cognitive preparation and exposure and were included in the total of 20 sessions offered. If a double session took place, data were recorded for only one of the two sessions, as patients completed the questionnaire only once per appointment. Out of the 1623 sessions conducted, 290 (18%) were double sessions. All patients, except for one, participated in at least one double session.

### 2.2 Assessments

Before each session, 12 items from the self‐report Eating Disorder Examination‐Questionnaire (EDE‐Q) (Fairburn and Beglin 1994; Hilbert and Tuschen‐Caffier 2016) were assessed for the preceding 7 days.

#### 2.2.1 Overvaluation of Shape and Weight

The “importance of weight” and “importance of shape” items of the EDE‐Q were combined into a single item (Grilo et al. 2010). Patients were asked whether their body shape or weight affected how they thought about themselves. Overvaluation of shape and weight was evaluated on a 7‐point Likert scale ranging from 0 (“not at all”) to 6 (“extremely”) with higher scores indicating greater overvaluation of shape and weight.

#### 2.2.2 Dietary Restraint

In order to assess dietary restraint, the EDE‐Q item “restraint over eating” was used. Participants were asked how many of the past 7 days they had deliberately tried to limit the amount of food they ate to affect their shape or weight, regardless of whether they were successful. A higher number of days indicated greater dietary restraint.

#### 2.2.3 Objective Binge Eating

To measure OBEs, the binge‐eating item of the EDE‐Q was used. Patients were asked how many of the past 7 days they had eaten an unusually large amount of food and felt that they had lost control over their eating behavior.

### 2.3 Data Analysis

Data analysis was performed using R (R Core Team 2023). As the process‐related EDE‐Q assessed symptoms over the past 7 days, only sessions that were spaced at least 7 days apart were included to ensure consistency and avoid overlapping data. Consequently, 398 of the initial 1334 data were excluded, as some patients attended multiple sessions per week during the first month of treatment, resulting in a final sample of 936 sessions for analysis. Paired *t*‐tests were used to analyze whether overvaluation of shape and weight, dietary restraint, and OBEs decreased from the first to the last week of CBT. The proportion of total variance of overvaluation of shape and weight, dietary restraint, and OBEs attributable to within‐person and between‐person variance was examined by calculating the intraclass correlation. A low intraclass correlation, close to zero, indicated minimal between‐individual variance, potentially making multilevel analysis unnecessary.

Dynamic structural equation modeling (DSEM) was used (Asparouhov et al. 2018) to examine the temporal relationship between overvaluation of shape and weight, dietary restraint, and OBEs. DSEM was implemented using Mplus v.8.7 (Muthén and Muthén 2017). Weeks were treated as a time variable, and data from a minimum of five patients per week were required to ensure reliable estimates. Thus, 24 out of 26 weeks were used. DSEM combines elements of time series analysis, multilevel modeling, and structural equation modeling (Hamaker et al. 2021). It is suitable for moderate sample sizes and time points (Rubel et al. 2019; Schuurman et al. 2016). DSEM integrates multiple steps into a single model (Hamaker et al. 2021; Rubel et al. 2019): It decomposes observed data for each individual (*i*) at a given time (*t*), such as OBEs_it, into within‐person and between‐person scores. A latent mean representing each patient across all time points captures between‐person variability (i.e., differences between individuals at a single time point), interpreted as a trait score, while deviations from the latent mean represent the session‐specific within‐person variability (i.e., fluctuations within an individual over time) (Hamaker et al. 2021). Missing data and model parameters were estimated with the Markov Chain Monte Carlo (MCMC) algorithm. This approach does not exclude participants with incomplete data; instead, MCMC iteratively samples from the posterior distributions, treating missing observations as additional parameters (Asparouhov et al. 2018).

**FIGURE 1:**
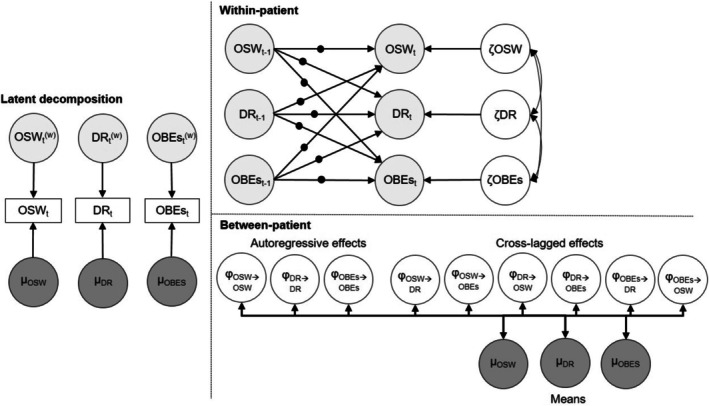
Dynamic structural equation model (DSEM) of overvaluation of shape and weight (OSW), dietary restraint (DR), and objective binge‐eating episodes (OBEs), adapted from Hamaker et al. (Hamaker et al. 2021). The left panel presents the decomposition into within‐patient and between‐patient components. The top right panel shows the within‐patient level model, including the random effects marked as black dots, and the bottom right panel shows the between‐patient level model.

Figure 1 presents the DSEM of overvaluation of shape and weight, dietary restraint, and OBEs. Within‐person standardized DSEM estimates allowed interpretation of the cross‐lagged associations (Schuurman et al. 2016). Convergence of the models was confirmed by inspecting proportional scale reduction (PSR) values and trace plots (Asparouhov et al. 2018). The Mplus code is included in the supplement (Figure S1).

In addition, two sensitivity analyses of the DSEM were conducted. First, considering that gender was meta‐analytically found to moderate treatment outcomes in BED (Hilbert et al. 2019), the DSEM was rerun exclusively with women (85.71% of our sample) to examine the consistency of results within this subgroup. Second, given the correlated nature of overvaluation of shape and weight and dietary restraint and potentially different temporal patterns, separate DSEMs including only one mediator at once were conducted. Alongside a better understanding of the unique contributions of each mediator within CBT for patients with BED, these separate models allow for robustness checks of the main analysis.

## 3 Results

### 3.1 Descriptive Statistics and Overall Change of Overvaluation of Shape and Weight, Dietary Restraint, and OBEs

**FIGURE 2:**
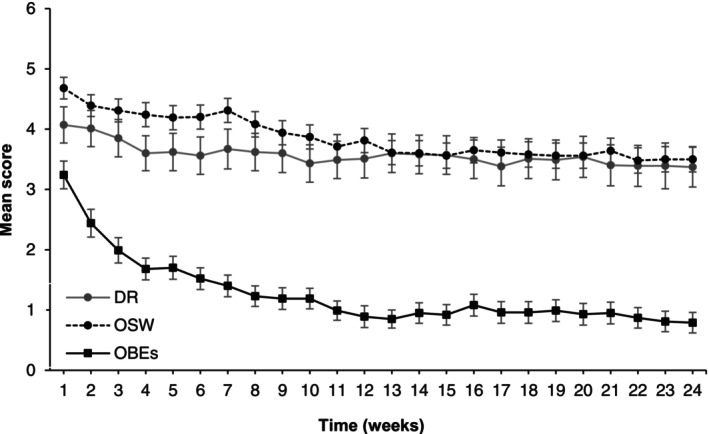
Mean values of overvaluation of shape and weight (OSW), dietary restraint (DR), and objective binge‐eating episodes (OBEs) over 24 weeks of cognitive‐behavioral therapy. Data from weeks with < 5 patients were excluded. Missing data were handled using the last observation carried forward (LOCF) method. Error bars indicate the standard error of the mean.

Mean levels of overvaluation of shape and weight, dietary restraint, and OBEs were 3.94 (SD = 1.87), 3.62 (SD = 2.90), and 1.48 (SD = 1.84) across all treatment weeks. Overvaluation of shape and weight decreased significantly from the first week (*M* = 4.68, SD = 1.68) to the last (*M* = 3.54, SD = 1.95), *t*(83) = 5.64, *p* < 0.001, *d* = 0.63, indicating a medium effect. Dietary restraint significantly decreased from the first (*M* = 4.11, SD = 3.04) to the last week (*M* = 3.41, SD = 3.04), *t*(82) = 2.00, *p* = 0.048, *d* = 0.24, with a small effect. OBEs decreased significantly from the first (*M* = 3.24, SD = 2.07) to the last week (*M* = 0.81, SD = 1.54), *t*(83) = 9.75, *p* < 0.001, *d* = 1.33, with a large effect. Change in mean levels is shown in Figure 2 and Table S1. The intraclass correlation analysis indicated that 68% of the variance in overvaluation of shape and weight, 62% in dietary restraint, and 36% in OBEs could be attributed to differences between patients.

### 3.2 Dynamic Structural Equation Modeling Analysis

There were significant autoregressive effects for overvaluation of shape and weight and OBEs in the DSEM indicating carryover effects from week to week for each variable (see Table 1). Overvaluation of shape and weight predicted next‐week OBEs, and dietary restraint predicted next‐week overvaluation of shape and weight. The cross‐lagged effects from dietary restraint to OBEs, from OBEs to dietary restraint and overvaluation of shape and weight, and from overvaluation of shape and weight to dietary restraint were not statistically significant.

**TABLE 1:** Fixed and random effects of DSEM for OSW, DR, and OBEs.

Parameters	WP‐standardized fixed effects		Random effects	
	Estimate (β)	95% credible interval	Estimate (variance)	95% credible interval
Intercepts/means				
μ_OSW_	**1.91**	**[1.44; 2.42]**	3.85	[2.56; 6.13]
μ_DR_	**1.17**	**[0.85; 1.51]**	8.92	[6.00; 13.88]
μ_OBEs_	**1.16**	**[0.74; 1.68]**	0.81	[0.37; 1.68]
Autoregressive effects				
ϕ_OSOS_: OSW_t‐1_ ➔ OSW_t_	**0.24**	**[0.16; 0.32]**	0.17	[0.10; 0.28]
ϕ_DRDR_: DR_t‐1_ ➔ DR_t_	**0.23**	**[0.15; 0.31]**	0.17	[0.10; 0.28]
ϕ_OBOB_: OBEs_t‐1_ ➔ OBEs_t_	**0.35**	**[0.27; 0.43]**	0.10	[0.05; 0.18]
Cross‐lagged effects				
ϕ_OSOB_: OSW_t‐1_ ➔ OBEs_t_	**0.14**	**[0.06; 0.23]**	0.05	[0.02; 0.11]
ϕ_OSDR_: OSW_t‐1_ ➔ DR_t_	0.02	[−0.07; 0.11]	0.03	[0.01; 0.07]
ϕ_DROB_: DR_t‐1_ ➔ OBEs_t_	−0.03	[−0.11; 0.06]	0.43	[0.24; 0.75]
ϕ_DROB_: DR_t‐1_ ➔ OSW_t_	**0.10**	**[0.01; 0.18]**	0.25	[0.12; 0.51]
ϕ_OBOS_: OBEs_t‐1_ ➔ OSW_t_	0.13	[−0.01; 0.24]	0.38	[0.16; 0.74]
ϕ_OBDR_: OBEs_t‐1_ ➔ DR_t_	0.01	[−0.10; 0.11]	0.10	[0.03; 0.24]

*Note*: Significant estimates of the fixed effects are bold.

Abbreviations: DR = dietary restraint; DSEM, dynamic structural equation model; OBEs, objective binge‐eating episodes; OSW, overvaluation of shape and weight.

The model explained 37% of the within‐person variance for overvaluation of shape and weight (95% CI [0.30, 0.43]), 40% of the within‐person variance for dietary restraint (95% CI [0.34, 0.46]), and 46% of the within‐person variance for OBEs (95% CI [0.38, 0.52]). The sensitivity analysis replicating the DSEM for women only and one mechanism at a time yielded similar results (see Tables S2–S4). In the women‐only analysis, an additionally significant cross‐lagged effect from OBEs to overvaluation of shape and weight was found (*β* = 0.15, 95% CI [0.02; 0.25]).

## 4 Discussion

The present study contributes to the growing field of mechanism research in psychotherapy by examining specific processes of change in CBT for BED. Through session‐by‐session assessments of two proposed mechanisms—overvaluation of shape and weight and dietary restraint—derived from the cognitive‐behavioral maintenance model of BED (Fairburn et al. 2003), this study enabled a fine‐grained, temporal analysis of the dynamic relationship between these mechanisms and symptom change during treatment. In a sample of 84 adults with BED receiving CBT, dynamic structural equation modeling (DSEM) revealed a significant within‐person effect of overvaluation of shape and weight on subsequent OBEs, while dietary restraint showed no direct effect on subsequent OBEs. Specifically, it was shown that weeks characterized by lower levels of overvaluation were followed by fewer OBEs. Although dietary restraint was not directly associated with reductions in OBEs, it was prospectively linked to fluctuations in overvaluation: weeks with higher dietary restraint levels predicted higher overvaluation of shape and weight in the following week. This result suggests that dietary restraint contributes to changes in OBEs indirectly or via synergistic pathways involving other processes. Taken together, the findings underscore the clinical importance of addressing both overvaluation of shape and weight and dietary restraint, which are not only central maintenance factors of BED but also candidate mechanisms of change. The modification of these constructs may represent key pathways through which CBT exerts its therapeutic effect.

As hypothesized, overvaluation of shape and weight significantly improved from the start to the end of CBT, supporting prior evidence of reduced shape and weight concerns from pre‐ to post‐treatment in patients with BED receiving CBT (Carrard et al. 2011; Grilo et al. 2011). Significant week‐to‐week effects of overvaluation of shape and weight on OBEs were found, consistent with effects of overvaluation of shape and weight on binge eating observed during CBT in patients with BN (Srivastava et al. 2023a) and in line with available evidence on pre‐CBT overvaluation of shape and weight predicting remission at 12 months (Grilo et al. 2013) and 60‐week (Cooper et al. 2016) follow‐up in adults with BED. In the full sample, OBEs did not significantly affect subsequent overvaluation of shape and weight, aligning with findings from Srivastava et al. (Srivastava et al. 2023a), where changes in binge eating did not predict changes in overvaluation of shape and weight at the next session. However, a sensitivity analysis revealed a significant effect of OBEs on overvaluation of shape and weight for women, indicating a need to explore gender differences in the relationship between binge eating and body image during CBT. Absent effects of overvaluation of shape and weight on subsequent dietary restraint contradict the transdiagnostic cognitive‐behavioral maintenance model, originally developed for patients with BN (Fairburn et al. 2003), which proposes a vicious cycle where overvaluation of shape and weight leads to attempts to restrict food intake, and previous findings in patients with BN (Srivastava et al. 2023a). This discrepancy may be related to the finding that patients with BED showed lower dietary restraint compared to those with BN (Elran‐Barak et al. 2015).

With regard to dietary restraint, the present study is, to our knowledge, the first to specifically examine the relevance of session‐by‐session changes in dietary restraint for OBEs in individuals with BED. Across the treatment period, only small reductions in dietary restraint were observed, which is consistent with prior research (Grilo and Pittman 2024). Although cognitive‐behavioral models of BED propose a causal relationship between dietary restraint and binge eating, the present study did not reveal a direct temporal association between week‐to‐week changes in dietary restraint and subsequent changes in OBEs. This finding aligns with previous experimental and observational research suggesting that dietary restraint may function more as a moderating factor rather than as a direct driver of binge eating (Jansen 2025). Similarly, both laboratory‐based studies manipulating dietary restriction and EMA studies exploring day‐to‐day associations between dietary restriction and binge eating have failed to yield consistent evidence for a direct link between greater dietary restriction and more OBEs in treatment‐seeking samples with binge‐spectrum disorders (Bartholomay et al. 2024; Manasse et al. 2023).

Interestingly, while dietary restraint was not directly associated with subsequent OBEs, it was significantly related to overvaluation of shape and weight in the following week. This finding indicates that dietary restraint may indirectly contribute to binge eating via its influence on overvaluation, a pathway that is also consistent with findings in BN (Srivastava et al. 2023a). Jansen (Jansen 2025) has further proposed that dietary restraint may reflect underlying dysfunctional beliefs concerning shape, weight, and eating. Accordingly, targeting dietary restraint in CBT for BED may primarily function to reduce dysfunctional beliefs about shape and weight, which, in turn, may contribute to decreases in OBEs. The efficacy of CBT in reducing OBEs through interventions on the normalization of eating behavior—a central therapeutic component—may be less attributable to changes in dietary restraint per se and more to other mechanisms, such as regular eating patterns or reductions in food cravings (Sivyer et al. 2020). These alternative mechanisms warrant further investigation.

Notably, one important conceptual distinction that was beyond the scope of the present study concerns the differentiation between flexible and rigid forms of dietary restraint. The single‐item measure applied in this study did not allow for such differentiation. Previous research suggested that rigid restraint—characterized by dichotomous, rule‐based eating patterns—is more closely associated with disordered eating behaviors, whereas flexible restraint reflects a more adaptive and sustainable approach to dietary regulation (Westenhoefer et al. 1999). Future studies employing more fine‐grained assessments will be needed to determine whether changes in flexible versus rigid restraint differentially predict treatment outcomes in CBT for BED (Grilo and Pittman 2024).

Strengths of this multi‐center study include the use of a clinical interview to assess BED, a standardized, manual‐based CBT (Hilbert and Tuschen‐Caffier 2010) with excellent adherence (Brauhardt et al. 2014), a longitudinal design with weekly assessments, and advanced statistical techniques to differentiate between within‐person and between‐person effects. Although interview‐based assessments would have further strengthened the validity of the process measures, their implementation was not feasible due to concerns about patient burden and the lack of blinded assessors at each session. Therefore, self‐report measures were employed, using carefully selected items from the well‐established EDE‐Q, adapted to assess changes in binge eating and eating disorder psychopathology over the past 7 days. Especially for dietary restraint, the use of a single item represents a limitation, as it did not allow for differentiation between distinct subtypes of restraint, such as rigid and flexible control (Westenhoefer et al. 1999). A further limitation concerns the exclusion of approximately 30% of the initial sessions, as only sessions that were spaced at least 7 days apart were included to avoid overlap in the reference period of the self‐report measures. Although this exclusion was necessary to maintain the temporal consistency required for meaningful analyses, it may have affected the stability and generalizability of the findings. Additionally, although the study's exclusion criteria were intentionally kept minimal compared to other RCTs for BED (Hilbert et al. 2019), some individuals, for example, patients whose body mass index (BMI, kg/m^2^) fell outside the predefined range, were not eligible for participation. This may have limited the representativeness of the sample in relation to the broader population of adults with BED. In terms of treatment changes, Figure 2 shows that changes mainly occurred within the first 2 months of therapy. Due to methodological limitations, it was not possible to determine when the most significant changes took place, which should be investigated in future studies.

The present study advances our understanding of CBT for BED by showing that overvaluation of shape and weight predicted subsequent OBEs during CBT, while dietary restraint did not directly, but indirectly through effects on overvaluation of shape and weight have an impact on OBEs. The present study thus highlights the clinical importance of addressing both overvaluation of shape and weight and dietary restraint in CBT for BED. To further optimize treatment efficacy, it will be essential to determine the most appropriate timing for interventions targeting these processes. Moreover, exploring potential synergistic effects between different treatment components could offer valuable insights for enhancing therapeutic outcomes. In this context, the application of multicomponent and factorial intervention designs will be critical for advancing the understanding of change mechanisms in CBT for BED and for informing clinical practice (Manasse et al. 2019). To further unravel the “why” behind psychotherapy's success (Kazdin 2007), future studies should combine session‐wise assessments of outcomes with a broader range of potential mechanisms of change, such as food reward sensitivity, emotion regulation, and inhibitory control (Schaefer et al. 2023). Additionally, the use of more ecologically valid assessment methods, for example via EMA (Lorenzo‐Luaces et al. 2015), may capture change processes more accurately and in real‐time. In parallel, advancing our understanding of the “how” behind CBT's efficacy for BED will require a more detailed examination of the relationship between active treatment elements—including the delivery of interventions (i.e., therapist adherence (Puls et al. 2019)), the patient's understanding of the delivered content (receipt), and the application of learned skills in daily life (transfer, e.g., homework completion (Srivastava et al. 2023b))—and their role in driving changes in both mediators and clinical outcomes (Cohen et al. 2023).

## Author Contributions

**Ricarda Schmidt:** conceptualization, investigation, methodology, resources, supervision, writing – review and editing. **Danielle Schewe:** conceptualization, formal analysis, visualization, writing – original draft, writing – review and editing. **Stephan Herpertz:** investigation, resources, writing – review and editing. **Stephan Zipfel:** investigation, resources, writing – review and editing. **Brunna Tuschen‐Caffier:** investigation, resources, writing – review and editing. **Hans‐Christoph Friederich:** investigation, resources, writing – review and editing. **Andreas Mayr:** investigation, methodology, resources, writing – review and editing. **Martina de Zwaan:** funding acquisition, investigation, project administration, resources, writing – review and editing. **Anja Hilbert:** conceptualization, funding acquisition, investigation, project administration, resources, supervision, writing – review and editing.

## Conflicts of Interest

Dr. Hilbert reports receiving royalties for books on the treatment of eating disorders and obesity with Hogrefe and Kohlhammer; honoraria for workshops and lectures on eating disorders and obesity and their treatment, including from Lilly and Novo Nordisk; honoraria as editor of the *International Journal of Eating Disorders* and the journal *Psychotherapeut*; honoraria as a reviewer from Oxford University Press and the German Society for Nutrition; and honoraria as a consultant for WeightWatchers, Zweites Deutsches Fernsehen, and Takeda. Dr. Herpertz reports giving lectures supported by travel grants from Berlin Chemie, Lilly, and Sanofi and authoring books and articles published by Springer and Thieme. Dr. Zipfel reports publishing books and articles on the psychotherapy of eating disorders, available through Thieme, Springer, Routledge, and Elsevier. Dr. Tuschen‐Caffier reports conducting lectures and workshops on eating disorders and having publications with Beltz, DGVT, Huber, Hogrefe, Kohlhammer, Psychosozial‐Verlag, and Wiley. Dr. Friederich reports presenting on eating disorders and has books and articles with Springer, Elsevier, Routledge, and Hogrefe. Dr. Mayr reports authoring articles and books through Thieme, Springer, Wiley, and Elsevier, and conducts statistics workshops for Siemens Healthcare. Dr. de Zwaan reports receiving grants from Novo Nordisk, Chiesi, and Novartis and authoring articles and books published by Springer, Routledge, and Elsevier. No other disclosures are reported, and the authors declare no conflicts of interest.

## Supporting information


**Data S1.** Supporting Information.

## Data Availability

Data availability is subject to reasonable request and can be obtained by contacting Prof. Dr. Hilbert (anja.hilbert@medizin.uni-leipzig.de).
