# Supplementary material for: Mechanisms of Change in Cognitive‐Behavioral Therapy for Adults With Binge‐Eating Disorder: A Dynamic Structural Equation Modeling Approach
Source: Int J Eat Disord. 2025 May 21;58(9):1701–9. doi: 10.1002/eat.24469 (PMC12423583; doi:10.1002/eat.24469)
Supplement: Supplementary file 1 — Data S1. Supporting Information. [file EAT-58-1701-s001.docx]

**Online Supplementary Material for:**

Ricarda Schmidt, Danielle Schewe, Stephan Herpertz, Stephan Zipfel, Brunna Tuschen-Caffier, Hans-Christoph Friederich, Andreas Mayr, Martina de Zwaan, Anja Hilbert. Mechanisms of change in cognitive-behavioral therapy for adults with binge-eating disorder: A dynamic structural equation modeling approach.

**Supplementary Tables**

**Supplementary Table S1.** Descriptive statistics

**Supplementary Table S2.** Fixed and random effects of DSEM for OSW, DR, and OBEs, women only

**Supplementary Table S3***.* Fixed and random effects of DSEM for OSW and OBEs

**Supplementary Table S4***.* Fixed and random effects of DSEM for OSW and DR

**Supplementary Figures**

**Supplementary Figure S1.** Mplus Code for the DSEM of OSW, DR, and OBEs.

**Table S1***:* Descriptive statistics

|  | **DR** | | | **OSW** | | | | **OBEs** | | |
| --- | --- | --- | --- | --- | --- | --- | --- | --- | --- | --- |
| **Week** | ***n*** | ***M*** | ***SD*** | | ***n*** | ***M*** | ***SD*** | ***n*** | ***M*** | ***SE*** |
| 1 | 83 | 4.11 | 2.73 | | 84 | 4.68 | 1.68 | 84 | 3.24 | 2.07 |
| 2 | 75 | 4.00 | 2.86 | | 77 | 4.47 | 1.67 | 76 | 2.25 | 1.95 |
| 3 | 68 | 3.88 | 2.77 | | 70 | 4.24 | 1.77 | 67 | 2.00 | 1.95 |
| 4 | 62 | 3.52 | 2.65 | | 62 | 4.27 | 1.76 | 63 | 1.65 | 1.55 |
| 5 | 63 | 4.00 | 2.96 | | 65 | 4.09 | 1.86 | 65 | 1.66 | 1.82 |
| 6 | 57 | 3.79 | 2.87 | | 59 | 4.27 | 1.85 | 58 | 1.64 | 1.76 |
| 7 | 52 | 3.85 | 2.7 | | 53 | 4.23 | 1.74 | 51 | 1.59 | 1.75 |
| 8 | 61 | 3.36 | 2.85 | | 63 | 4.08 | 1.96 | 62 | 1.34 | 1.66 |
| 9 | 51 | 3.2 | 2.86 | | 51 | 3.78 | 1.8 | 50 | 1.12 | 1.59 |
| 10 | 53 | 3.3 | 3.03 | | 54 | 3.52 | 1.88 | 53 | 1.09 | 1.57 |
| 11 | 55 | 3.45 | 2.92 | | 57 | 3.37 | 1.86 | 55 | 0.82 | 1.39 |
| 12 | 58 | 3.5 | 2.88 | | 58 | 3.67 | 1.86 | 55 | 1.02 | 1.79 |
| 13 | 50 | 3.04 | 2.94 | | 51 | 3.25 | 2.01 | 50 | 0.92 | 1.44 |
| 14 | 41 | 3.98 | 2.88 | | 40 | 3.70 | 1.88 | 41 | 1.17 | 1.80 |
| 15 | 44 | 3.18 | 3.04 | | 45 | 3.29 | 1.93 | 43 | 0.72 | 1.26 |
| 16 | 38 | 3.89 | 3.00 | | 39 | 3.72 | 1.97 | 37 | 1.32 | 1.70 |
| 17 | 36 | 3.08 | 3.07 | | 37 | 3.35 | 1.98 | 35 | 0.94 | 1.78 |
| 18 | 32 | 4.31 | 3.00 | | 32 | 3.59 | 1.76 | 33 | 0.97 | 1.42 |
| 19 | 22 | 3.09 | 3.12 | | 23 | 3.35 | 2.12 | 21 | 0.67 | 1.65 |
| 20 | 29 | 2.62 | 3.14 | | 29 | 4.00 | 1.96 | 29 | 0.93 | 1.79 |
| 21 | 20 | 3.25 | 3.26 | | 20 | 3.35 | 1.87 | 19 | 1.42 | 2.22 |
| 22 | 15 | 4.27 | 2.99 | | 15 | 4.53 | 1.6 | 14 | 0.86 | 1.10 |
| 23 | 14 | 2.86 | 3.16 | | 14 | 4.07 | 1.86 | 14 | 1.21 | 2.26 |
| 24 | 12 | 4.25 | 2.93 | | 12 | 4.25 | 1.86 | 11 | 0.82 | 1.33 |
| 25 | 1 | 3.00 | 0.00 | | 1 | 5.00 | 0.00 | 1 | 2.00 | 0.00 |
| 26 | 2 | 3.50 | 4.95 | | 2 | 5.00 | 1.41 | 2 | 2.00 | 2.83 |

*DR = Dietary restraint; OSW = Overvaluation of weight and shape; OBEs = Objective binge-eating episodes.*

**Table S2***:* Fixed and random effects of DSEM for OSW, DR, and OBEs, women only

|  | **WP-standardized fixed effects** | | **Random effects** | | |
| --- | --- | --- | --- | --- | --- |
| **Parameters** | **Estimate (β)** | **95% credible interval** | | **Estimate (variance)** | **95% credible interval** |
| Intercepts/means |  |  | |  |  |
| μ_OSW_ | **1.94** | **[1.43; 2.50]** | | 3.99 | [2.60; 6.57] |
| μ_DR_ | **1.14** | **[0.79; 1.52]** | | 9.22 | [5.92; 15.09] |
| μ_OBEs_ | **1.01** | **[0.55; 1.52]** | | 1.28 | [0.45; 2.73] |
| Autoregressive effects |  |  | |  |  |
| φ_OSOS_: OSW_t-1_ 🡪 OSW_t_ | **0.23** | **[0.14; 0.32]** | | 0.21 | [0.12; 0.36] |
| φ_DRDR_: DR_t-1_ 🡪 DR_t_ | **0.24** | **[0.16; 0.33]** | | 0.18 | [0.10; 0.32] |
| φ_OBOB_: OBEs_t-1_ 🡪 OBEs_t_ | **0.38** | **[0.29; 0.46]** | | 0.11 | [0.05; 0.20] |
| Cross-lagged effects |  |  | |  |  |
| φ_OSOB_: OSW_t-1_ 🡪 OBEs_t_ | **0.15** | **[0.06; 0.24]** | | 0.05 | [0.02; 0.11] |
| φ_OSDR_: OSW_t-1_ 🡪 DR_t_ | 0.00 | [-0.09; 0.09] | | 0.03 | [0.01; 0.08] |
| φ_DROB_: DR_t-1_ 🡪 OBEs_t_ | -0.01 | [-0.09; 0.12] | | 0.44 | [0.24; 0.80] |
| φ_DROB_: DR_t-1_ 🡪 OSW_t_ | **0.10** | **[0.01; 0.20]** | | 0.29 | [0.13; 0.63] |
| φ_OBOS_: OBEs_t-1_ 🡪 OSW_t_ | **0.15** | **[0.01; 0.25]** | | 0.43 | [0.17; 0.90] |
| φ_OBDR_: OBEs_t-1_ 🡪 DR_t_ | 0.01 | [-0.09; 0.12] | | 0.07 | [0.02; 0.19] |

*DSEM = Dynamic structural equation model; OSW = Overvaluation of shape and weight,
DR = Dietary restraint, OBEs = Objective binge-eating episodes. Significant estimates of the fixed effects are bold.*

**Table S3***:* Fixed and random effects of DSEM for OSW and OBEs

|  | **WP-standardized Fixed effects** | | **Random effects** | | |
| --- | --- | --- | --- | --- | --- |
| **Parameters** | **Estimate (β)** | **95% credible interval** | | **Estimate (variance)** | **95% credible interval** |
| Intercepts/means |  |  | |  |  |
| μ_OSW_ | **2.08** | **[1.58; 2.62]** | | 3.18 | [2.17; 4.87] |
| μ_OBEs_ | **1.53** | **[1.00; 2.26]** | | 0.45 | [0.19; 1.00] |
| Autoregressive effects |  |  | |  |  |
| φ_OSOS_: OSW_t-1_ 🡪 OSW_t_ | **0.30** | **[0.22; 0.39]** | | 0.12 | [0.07; 0.21] |
| φ_OBOB_: OBEs_t-1_ 🡪 OBEs_t_ | **0.46** | **[0.38; 0.55]** | | 0.07 | [0.03; 0.13] |
| Cross-lagged effects |  |  | |  |  |
| φ_OSOB_: OSW_t-1_ 🡪 OBEs_t_ | **0.16** | **[0.07; 0.24]** | | 0.03 | [0.01; 0.07] |
| φ_OBOS_: OBEs_t-1_ 🡪 OSW_t_ | **0.12** | **[0.02; 0.22]** | | 0.16 | [0.04; 0.38] |

*DSEM = Dynamic structural equation model; OSW = Overvaluation of shape and weight,
DR = Dietary restraint, OBEs = Objective binge-eating episodes. Significant estimates of the fixed effects are bold.*

**Table S4***:* Fixed and random effects of DSEM for OSW and DR

|  | **WP-standardized Fixed effects** | | **Random effects** | | |
| --- | --- | --- | --- | --- | --- |
| **Parameters** | **Estimate (β)** | **95% credible interval** | | **Estimate (variance)** | **95% credible interval** |
| Intercepts/means |  |  | |  |  |
| μ_DR_ | **1.31** | **[0.99; 1.66]** | | 7.39 | [5.08; 11.05] |
| μ_OBEs_ | **1.51** | **[1.00; 2.21]** | | 0.54 | [0.21; 1.11] |
| Autoregressive effects |  |  | |  |  |
| φ_DRDR_: DR_t-1_ 🡪 DR_t_ | **0.26** | **[0.17; 0.34]** | | 0.12 | [0.07; 0.20] |
| φ_OBOB_: OBEs_t-1_ 🡪 OBEs_t_ | **0.49** | **[0.40; 0.56]** | | 0.05 | [0.02; 0.09] |
| Cross-lagged effects |  |  | |  |  |
| φ_DROB_: DR_t-1_ 🡪 OBEs_t_ | 0.03 | [-0.06; 0.10] | | 0.24 | [0.10; 0.45] |
| φ_OBDR_: OBEs_t-1_ 🡪 DR_t_ | -0.004 | [-0.10; 0.11] | | 0.05 | [0.01; 0.15] |

*DSEM = Dynamic structural equation model; OSW = Overvaluation of shape and weight,*

*DR = Dietary restraint, OBEs = Objective binge-eating episodes. Significant estimates of the fixed effects are bold.*

**Figure S1***:* Mplus Code for the DSEM of OSW, DR, and OBEs. **
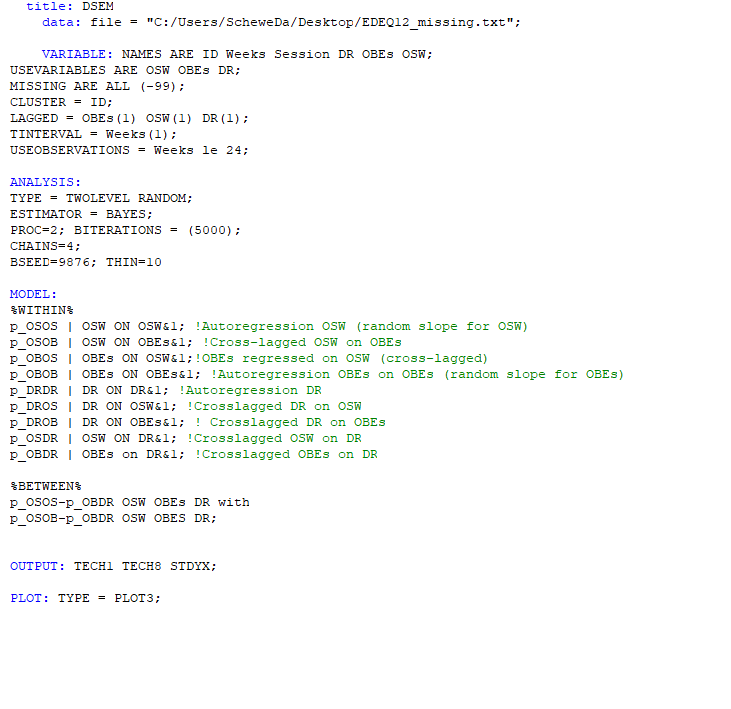
**
